# Patient-controlled subcutaneous analgesia with hydromorphone in cancer pain management

**DOI:** 10.3389/fpain.2025.1674672

**Published:** 2025-12-17

**Authors:** Zhongkai Wang, Pengqing Jiao

**Affiliations:** 1 Department of Pain Medicine and Rehabilitation, The Fourth Hospital of Hebei Medical University, Shijiazhuang, Hebei, China; 2 Department of Rheumatology and Immunology, The Fourth Hospital of Hebei Medical University, Shijiazhuang, Hebei, China

**Keywords:** hydromorphone, subcutaneous injection, refractory cancer pain, pain score, anxiety, depression

## Abstract

**Background:**

Cancer pain is a significant public health concern worldwide, necessitating effective management strategies. This study aimed to explore the effects of patient-controlled subcutaneous analgesia (PCSA) with hydromorphone hydrochloride injection on refractory cancer pain.

**Methods:**

We conducted a retrospective observational study involving patients who received PCSA with hydromorphone hydrochloride injection at our hospital from December 2022 to May 2023. All patients in this study were initially hospitalized to undergo dose titration and safety assessment for subcutaneous hydromorphone PCSA, ensuring drug tolerance and stable pump operation. After achieving dose stabilization, most patients continued analgesic therapy at home using the pump, with dynamic monitoring and dose adjustments conducted via telephone follow-ups and outpatient visits. Pain was assessed using the numerical rating scale (NRS), while anxiety and depression were evaluated using the Edmonton Symptom Assessment Scale (ESAS-R) and sleep quality was measured with the Pittsburgh Sleep Quality Index (PSQI). The incidence of adverse drug reactions was also documented.

**Results:**

The treatment demonstrated significant improvement across all observed parameters. After the continuous subcutaneous injection of hydromorphone hydrochloride infusion for analgesia,the median NRS pain score decreased from 6 to 1 (*p* < 0.0001), the mean sleep quality score decreased from 13.85 to 7.488 (*p* < 0.0001), the median anxiety state score decreased from 5 to 1 (*p* < 0.0001), the median depression score decreased from a baseline of 3 to 0.5 (*p* < 0.0001), and the median equivalent oral morphine dose decreased from 140 mg to 52 mg (*p* < 0.0001).

**Conclusions:**

PCSA with hydromorphone hydrochloride injection offers significant therapeutic benefits for refractory cancer pain. It effectively reduces pain intensity, decreases opioid dosage, mitigates certain adverse reactions, and is associated with reduced anxiety and depression as well as improved sleep quality.

## Introduction

The prognosis of many types of cancer is improving each year, meaning that the prevalence of patients with cancer is increasing (1–3), leading to an increase in the frequency of survivorship issues (3, 4). With the increasing number of cancer cases, cancer pain has become one of the most important public health concerns worldwide, with a prevalence of about 45% and a poor pain control rate of 40% (5, 6). In China, a standardized treatment of cancer pain has been promoted extensively, leading to effective relief of pain symptoms in 80%–90% of the patients, but refractory pain is still observed in 10%–20% of the patients (7). A conventional oral drug treatment regimen is less effective and may cause intolerable adverse reactions in patients with cancer pain (6). Currently, there is an absence of a globally standardized definition and diagnostic criteria for refractory cancer pain (8, 9). According to the 2017 Chinese Expert Consensus on the Diagnosis and Treatment of Refractory Cancer Pain, it is defined as moderate to severe pain caused by the tumor itself or tumor-related treatment factors, wherein the patient's pain remains inadequately relieved after 1–2 weeks of standardized pharmacotherapy and/or the adverse effects are intolerable, and its diagnosis requires meeting both of the following criteria simultaneously: persistent pain with a Numeric Rating Scale (NRS) score ≥4 and/or breakthrough pain occurring ≥3 times per day, along with inadequate pain relief after 1–2 weeks of treatment conducted in accordance with relevant guidelines using opioids alone and/or combined with adjuvant analgesics, and/or the emergence of intolerable adverse reactions. Indeed, refractory cancer pain leads to impaired physical and mental functions, decreased productivity, anxiety, stress, depression, and poor sleep quality, leading to an overuse of healthcare resources (10). Optimizing cancer pain management would be associated with healthcare cost savings (11). Consequently, managing refractory cancer pain remains a significant challenge in comprehensive cancer care (10). One reason for pain refractoriness is that patients not only experience pain but also multiple other psychological and social issues (6, 10). Therefore, optimal pain control strategies are necessary to reduce the personal, familial, and societal burden of refractory cancer pain.

This drug delivery method can stabilize the blood drug concentration in patients while achieving patient-controlled analgesia (PCA) (12). Hydromorphone is a pure opioid receptor agonist and a semisynthetic morphine derivative. Although its chemical structure is similar to that of morphine, hydromorphone has a ketone group at position 6 and hydrogenated double bonds at positions 7 and 8. These slight differences make the analgesic effect of hydromorphone 5–10 times that of morphine and facilitate the penetration of the blood-brain barrier to exert its effects (13, 14). Additionally, the lipid solubility of hydromorphone is 10 times that of morphine, making it more suitable for subcutaneous injection than morphine (15–17). Hydromorphone exhibits both high lipid solubility and favorable water solubility. Its lipid solubility is greater than that of morphine, facilitating subcutaneous tissue absorption and rapid onset of action. Simultaneously, its water solubility surpasses that of fentanyl and sufentanil, enabling stable dissolution at higher concentrations. This unique property grants hydromorphone a more ideal balance between lipid and water solubility compared to other opioids, making it suitable for preparing small-volume, long-term stable subcutaneous injections, which reduces infusion volume and minimizes risks of precipitation or local irritation (18). Still, its use for patient-controlled subcutaneous analgesia (PCSA) remains to be thoroughly investigated in patients with refractory cancer pain.

Therefore, this study aimed to evaluate the effect of PCSA with hydromorphone hydrochloride injection on patients with refractory cancer pain. By quantifying the effects on pain, mood, and sleep, this study provides crucial clinical data on the utility of this approach for a challenging patient population.

## Materials and methods

### Study design and population

This retrospective observational study included 40 patients who received PCSA with hydromorphone hydrochloride injection from the Department of Pain Medicine and Rehabilitation of our hospital between December 2022 and May 2023. Table 1 shows the patients' basic information. The date when data access for the research began was 16/9/2023.The inclusion criteria were (1) ≥18 years of age, (2) pathological evidence of malignant tumor, (3) meeting the diagnostic criteria for refractory cancer pain before the administration of PCSA with hydromorphone hydrochloride injection (19), (4) no mental or consciousness disorders, and (5) expected survival of >1 month when starting PCSA. This study was approved by the Ethics Committee of our hospital. This article is a retrospective study. Therefore, our hospital's Ethics Committee has waived the requirement to obtain written informed consent from patients for participation in clinical research.

**Table 1:** Characteristics of the patients.

Characteristics		*n* (%)
Sex	Male	22 (55%)
	Female	18 (45%)
Age (years)		
All	62 (54.5, 69)	40 (100%)
≤65	56 (48.5, 60.5)	25 (62.5%)
>65	71 (68, 77)	15 (37.5%)
Tumor type	Lung cancer	8 (20%)
	Malignant tumor of the digestive tract	23 (57.5%)
	Cervical and ovarian cancer	5 (12.5%)
	Other malignancies	4 (10%)
Reason for the application of subcutaneous analgesic pump	Poor effect of the original analgesic regimen	33 (82.5%)
	Intolerance to oral opioids	7 (17.5%)
Pain intensity	Moderate	27 (67.5%)
	Severe	13 (32.5%)

Numerical Rating Scale (NRS) Criteria: 0: No pain, 1–3: Mild pain, 4–6: Moderate pain, 7–10: Severe pain, 10: Worst possible pain.

### Data collection and definitions

Upon admission, the patient's analgesic treatment was converted into a subcutaneous hydromorphone dose based on their previous use of analgesics. Then, the background and single doses were adjusted according to the patient's numerical rating scale (NRS) score and the number of additional doses. The NRS scores of the patients on days 0, 1, 2, 3, 7, 15and 30 and their adverse reactions were recorded. Electrocardiogram monitoring was routinely performed on day 1 of PCSA to observe the indexes of heart rate, blood oxygen, respiratory rate, and blood pressure. All patients are to have their dose titration assessment completed within 3 days of admission. Following dose titration and stabilization, patients were discharged home with the pump for ongoing pain management. The NRS scores and anxiety and depression scores on days 7, 15, and 30 were compared. On day 30, the sleep status score was calculated. The Anxiety and Depression (ESAS-R) scale reflects the patient's immediate psychological state, which is considerably influenced by short-term changes in analgesia and overall comfort. To dynamically observe trends in psychological status, assessments were conducted before treatment and on days 7, 15, and 30 to document the psychological improvement process following both short-term and sustained analgesic effects. The Pittsburgh Sleep Quality Index (PSQI) assesses overall sleep quality over a “1-month” recall period. Consequently, PSQI evaluations were performed at baseline (prior to hydromorphone injection therapy) and on day 30 post-treatment to assess sleep quality.

The original solution of hydromorphone hydrochloride injection was used for preparing the subcutaneous analgesic pump. The time required to stabilize the drug concentration in the one-time storage box could reach 15 days; hence, we usually calculated the dose for 1 week during the first application and then calculated the dose for 2 weeks after achieving stable pain control.

As per routine care during the study period and due to drug stability issues, the pharmacists calculated the doses for 1 week at a time. According to the subcutaneous-to-oral conversion ratio of 1:2, the conversion relationship between hydromorphone and morphine was 1:10. The daily dose was divided by 24 to calculate the hourly dose of hydromorphone hydrochloride injection. Then, approximately two-thirds of the dose was used as the background dose, which was generally <2 mL/h to avoid local malabsorption. The rescue dose was 1–2 times the background dose, and the locking time was 20 min. Subcutaneously administered hydromorphone has an onset of action of approximately 10–15 min, with peak plasma concentrations typically occurring within 20–30 min and an elimination half-life of about 2.5–3 h (20). Based on the pharmacokinetic profile of hydromorphone, a lockout interval of 20 min not only covers its onset period but also prevents repeated button presses as the drug approaches its peak efficacy, representing an optimal balance between analgesic timeliness and safety. Multiple clinical studies recommend setting the lockout interval for opioid PCA within a range of 15–30 min, with 20 min being a commonly adopted median value in clinical practice. This setting helps maintain stable plasma drug concentrations while preserving patient autonomy, thereby reducing the risk of overdose and improving analgesic satisfaction (12, 20, 21). Depending on pain relief, the original background dose was maintained when the NRS score was <3, but it was increased by 25%–50% when the score was 4–6 and by 50%–100% when the score was >7. The dose was evaluated and adjusted again after 12 or 24 h until the effect was satisfactory.

All data were extracted from the medical charts based on the routine evaluations of patients on PCSA. As per routine practice for PCSA implementation during the study period, it was evaluated on days 0, 1, 2, 3, 7, 15 and 30. The anxiety and depression scores were evaluated on days 0, 7, 15, 30. On days 0 and 30, the sleep status score was calculated. The Edmonton Symptom Assessment Scale (ESAS-R) was used to evaluate their anxiety and depression states (22), and the Pittsburgh Sleep Quality Index (PSQI) was used to evaluate their sleep status (23). All scores were assessed according to the predetermined criteria. The incidence of adverse drug reactions was also extracted from the medical charts. The primary outcome was the change in NRS. The secondary outcomes were the changes in ESAS-R, PSQI, and equivalent morphine dose.

### Puncture method and site

We used a 24G or 26G indwelling needle for the puncture. Under strict aseptic conditions, the skin was disinfected, and the needle was placed at a subcutaneous puncture angle of <30° to avoid muscle layer penetration. For patients who were thin, the local skin could be pinched for puncture. The indwelling needle and infusion catheter were fixed appropriately with a transparent dressing, preferably for easy observation. Meanwhile, the retention date and time were recorded.

Multiple body sites were suitable for puncture, but the sites with good local circulation that do not affect the patient's activity or sleep were preferred. The commonly used area was the lower edge of the deltoid muscle in the upper arm. For patients under bed rest or with limited mobility, the medial or lateral thigh may be used.

### Statistical analysis

All the statistical analyses were performed using GraphPad Prism 9 and R programming language (version 4.2.1).For enumeration data, the chi-square test was used when the theoretical frequency was greater than 5, while fisher exact probability method was used when the theoretical frequency was less than or equal to 5. For quantitative data, Paired or unpaired *t*-tests were used for comparisons of two groups. For comparisons of three of more groups, one-way ANOVA was used. First, a normality test was performed. When the data were normally distributed, ANOVA was used. When the data did not conform to a normal distribution, the nonparametric test was used. Adjusted *p* values were used. According to the Shapiro–Wilk test, all scores except for the sleep score did not follow a normal distribution. The Friedman test was applied to evaluate changes in anxiety-depression scores and pain scores. *p* < 0.05 was considered to indicate statistical significance.

## Results

### Outcomes

**Figure 1:**
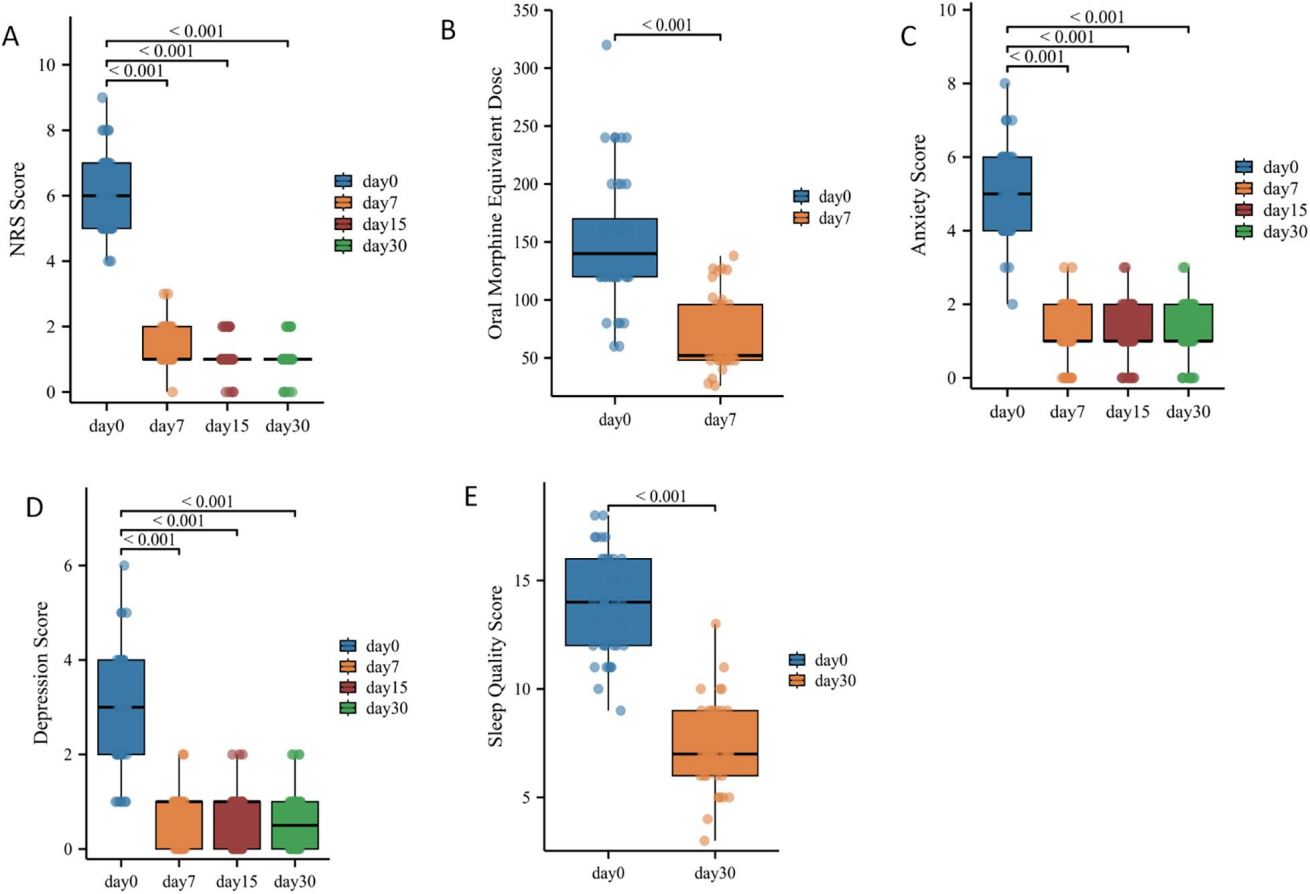
Changes in time of the various indicators before and after treatment. **(A)** numeric rating scale (NRS) score. **(B)** Oral morphine equivalent dose. **(C)** Anxiety score. **(D)** Depression score. **(E)** Sleep quality score.

After the continuous subcutaneous injection of hydromorphone hydrochloride infusion for analgesia,the median NRS pain score decreased from 6 to 1 (*p* < 0.0001), the mean sleep quality score decreased from 13.85 to 7.488 (*p* < 0.0001), the median anxiety state score decreased from 5 to 1 (*p* < 0.0001), the median depression score decreased from a baseline of 3 to 0.5 (*p* < 0.0001), and the median equivalent oral morphine dose decreased from 140 mg to 52 mg (*p* < 0.0001) (Table 2). The treatment demonstrated significant improvement across all observed parameters (Figure 1).

**Table 2:** Primary and secondary outcomes before and after treatment^d^.

Outcome	0 d	7 d	15 d	30 d	Test statistic *χ*^2^/*t*/*Z*	*p*-values
Primary outcome						
Pain intensity NRS					106.4	*P* < 0.001^a^
Median	6 (5, 7)	2 (2, 3)	1 (1, 2)	1 (1, 1)		
Secondary outcome						
Sleep quality					13.31	*P* < 0.001^c^
Mean	13.85 ± 2.34			7.68 ± 2.12		
Anxiety states					94.32	*P* < 0.001^a^
Median	5 (4, 6)	1 (1, 2)	1 (1, 2)	1 (1, 2)		
Depression states					80.42	*P* < 0.001^a^
Median	3 (2, 3)	1 (0, 1)	1 (0, 1)	0.5 (0, 1)		
Oral equivalent morphine doses					−4.263	*P* < 0.001^b^
Median	140 (120, 180)			52 (48, 96)		

a Friedman test the statistic is denoted by *χ*^2^.

b Wilcoxon signed-rank test the statistic is denoted by *Z*.

c Paired *t*-test the statistic is denoted by *t*.

d The “pre-treatment” adverse reaction data specifically refer to drug-related adverse reactions that occurred while patients were still on their previous oral or other opioid-based analgesic regimens, before switching to subcutaneous hydromorphone patient-controlled analgesia (PCSA). “0 d” represents pre-treatment data. The “post-treatment” data refer to newly emerged or persistent reactions during follow-up in patients using hydromorphone PCSA; 7 d, 15 d, and 30 d represent data for the corresponding indicators at the respective time points after treatment initiation.

## Adverse effects

Baseline adverse reactions are symptoms experienced by patients during their previous opioid treatment regimen. Compared with oral or topical opioids, the application of hydromorphone hydrochloride injection PCSA significantly reduced the frequencies of nausea and vomiting from 45% to 2.5% (*p* < 0.001) and constipation from 47.5% to 7.5% (*P* = 0.0324). There were no significant changes in dizziness (*P* = 0.0567) and pruritus (*P* = 0.1943) (Table 3).

**Table 3:** Comparison of side effects before and after treatment^b^.

Adverse events	0 d, *n* (%)	7 d, *n* (%)	15 d (%)	30 d, *n* (%)	*P* ^a^
Nausea and vomiting	18 (45.0%)	2 (5.0%)	1 (2.5%)	1 (2.5%)	<0.001
Constipation	19 (47.5%)	7 (17.5%)	4 (10%)	3 (7.5%)	0.0324
Dizziness	4 (10.0%)	1 (2.5%)	0	0	0.0567
Pruritus	3 (7.5%)	1 (2.5%)	0	0	0.1943

a All of the above statistical analyses were performed using Fisher's exact test.

b The “pre-treatment” adverse reaction data specifically refer to drug-related adverse reactions that occurred while patients were still on their previous oral or other opioid-based analgesic regimens, before switching to subcutaneous hydromorphone patient-controlled analgesia (PCSA). “0 d” represents pre-treatment data. The “post-treatment” data refer to newly emerged or persistent reactions during follow-up in patients using hydromorphone PCSA; 7 d, 15 d, and 30 d represent data for the corresponding indicators at the respective time points after treatment initiation.

## Discussion

This study found that PCSA with hydromorphone hydrochloride injection might have significant therapeutic advantages in refractory cancer pain by reducing pain, anxiety, depression, and poor sleep quality. Reductions in opioids and some adverse effects were also observed. The results suggest that PCSA with hydromorphone hydrochloride injection could be used to improve the management of patients with refractory cancer pain.

In the treatment of refractory cancer pain, switching opioid administration routes is common (24), and continuous subcutaneous administration is one of the most effective approaches for patients with severe pain requiring rapid analgesia (25). The primary outcome of the present study was the change in NRS after starting PCSA. The results showed a significant decrease in NRS scores after 7 days of PCSA in all patients. Patients were in a low-pain state on days 15 and 30. Those results are supported by previous studies (26–28). Indeed, a double-blind, randomized controlled trial showed that PCSA using hydromorphone significantly decreased the NRS (26). Of note, the trial observed no significant difference analgesic effects between hydromorphone and morphine (26). A prospective study by Xiao et al. (28) showed that PCSA using hydromorphone achieved a better analgesic effect than oral oxycontin. The present retrospective study also provides real-world evidence supporting the use of PCSA using hydromorphone hydrochloride for the management of refractory cancer pain.

Patients with cancer pain often suffer from anxiety, depression, and sleep disorders (29, 30). Arora et al. (29) reported that the pain intensity experienced by patients with cancer is closely related to the severity of anxiety and depression. In the present study, ESAS-R was used to determine the anxiety and depression scores of patients with moderate and severe cancer pain. Following the administration of hydromorphone hydrochloride injection PCSA, patients demonstrated a significant decrease in anxiety and depression scores from baseline levels, which were recorded during previous oral or topical opioid therapy. Moreover, patients with cancer pain often complain of poor sleep quality (31), and sleep quality improved significantly in patients with good pain control using PCSA and hydromorphone hydrochloride. This study demonstrates that improved pain control is associated with reductions in anxiety and depression as well as enhanced sleep quality (31).

This study employed a multi-timepoint assessment design, which was determined by the differences in temporal sensitivity across various observation indicators, resulting in distinct observation timepoints for different metrics. Anxiety and depression (ESAS-R) reflect immediate psychological states and are significantly influenced by short-term fluctuations in analgesic efficacy and comfort levels (32, 33). Therefore, anxiety and depression scores were assessed synchronously with pain scores at baseline (pre-treatment) and on days 7, 15, and 30 after initiation of hydromorphone hydrochloride injection PCSA therapy, to dynamically observe psychological improvement trends during early and sustained analgesia. Sleep quality was evaluated using the Pittsburgh Sleep Quality Index (PSQI). According to the original design of this scale (23), the PSQI assesses overall sleep quality “over the past month,” making repeated measurements within short periods (e.g., 7 days) potentially inaccurate due to overlapping reference periods. Out of respect for the scale's design integrity, we chose to conduct assessments at baseline and on day 30 after initiating hydromorphone hydrochloride injection PCSA therapy, to more scientifically reflect sleep improvements after reaching analgesia stabilization. The differences in assessment timepoints were deliberately established based on the characteristics of each indicator and the standardized design specifications of the respective scales to ensure methodological rigor and reliability.

Those advantages of PCSA could be related to the following aspects. First, PCSA can stabilize the blood drug concentration, improve drug bioavailability, and avoid the first-pass effect. Second, hydromorphone hydrochloride injection has better lipid solubility and absorption by the body than oral opioids. Hale et al. (34) showed in patients with low back pain that switching from morphine to hydromorphone significantly improved the pain intensity scores. Third, PCA technology enables patients to participate in pain management more actively and treat analgesia in a timelier manner than the conventional approach (17), increasing their empowerment and translating into better psychological outcomes (35). In addition, the reduction in the equivalent dose of oral morphine after the subcutaneous injection of hydromorphone hydrochloride was similar to the finding of Jackson et al. (36). Therefore, the initial dose can be better predicted when switching to the subcutaneous pumping route.

Patients using PCSA hydromorphone hydrochloride for analgesia were also less likely to experience nausea, vomiting, and constipation than when administered oral opioids. No other serious adverse reactions were observed, and the patients had better tolerance; thus, this drug delivery method is an excellent analgesic approach for relieving refractory cancer pain. It is important to emphasize that the improvement in adverse reactions observed in this study was not due to direct antiemetic or laxative effects of hydromorphone. This improvement was closely related to its pharmacological profile and the modality of subcutaneous continuous infusion. As a semisynthetic derivative of morphine, hydromorphone exhibits higher lipophilicity, greater receptor selectivity, and lower active metabolite accumulation, resulting in reduced stimulation of the central emetic zone (18). Furthermore, subcutaneous continuous infusion maintains stable plasma drug concentrations, avoiding the peak-trough fluctuations associated with oral administration, thereby minimizing excessive stimulation of central and gastrointestinal receptors. Additionally, subcutaneous administration bypasses first-pass gastrointestinal metabolism, further reducing gastrointestinal motility inhibition and local irritation (37).This favorable adverse effect profile is also supported by prospective studies of PCSA using hydromorphone hydrochloride in cancer patients (26, 28).

The present study has limitations. The sample size was limited because the patients were from a single center and a limited study period. This single-center study with a small sample size may lead to insufficient statistical power and measurement bias. The absence of a parallel control group limits the ability to control for confounding factors, and thus the conclusions require validation by prospective randomized controlled trials (RCTs). Differences in baseline data status may result in an overestimation of efficacy, necessitating a larger sample size for further verification. Additionally, as this study involved only short-term observation, it cannot evaluate long-term effects. The study period was mainly limited by the start of using PCSA at the authors' hospital. In addition, pain, anxiety, depression, and sleep quality can be influenced by the educational level of the patients and their family members (38), as well as by the support from the family members and the community; these differences were not accounted for in the present study.

In conclusion, PCSA with hydromorphone hydrochloride injection might effectively reduce patients' pain scores and improve sleep quality and anxiety and depression states in patients with refractory cancer pain. In this study, patients could continue analgesia at home after completing dose titration. This treatment strategy should have an important practical significance for improvingthe overall quality of life in patients with refractory cancer pain.

## Data Availability

The original contributions presented in the study are included in the article/Supplementary Material, further inquiries can be directed to the corresponding author.
